# Noncoding-RNA-Based Therapeutics with an Emphasis on Prostatic Carcinoma—Progress and Challenges

**DOI:** 10.3390/vaccines10020276

**Published:** 2022-02-11

**Authors:** Victor E. Nava, Pin-Yu Perera, Nirbhay Kumar, Maneesh Jain

**Affiliations:** 1 Pathology Service, Veterans Affair Medical Center, Washington, DC 20422, USA; Pin-Yu.Perera@va.gov; 2 Department of Pathology, The George Washington University, Washington, DC 20037, USA; 3 Department of Global Health, Milken Institute School of Public Health, The George Washington University, Washington, DC 20052, USA; nkumar@gwu.edu; 4 Department of Medicine, The George Washington University School of Medicine and Health Sciences, Washington, DC 20037, USA; Maneesh.Jain@va.gov; 5 Hematology/Oncology Service, Veterans Affair Medical Center, Washington, DC 20422, USA

**Keywords:** noncoding RNA, ncRNA therapeutics, ncRNA “vaccines”, prostatic carcinoma

## Abstract

Noncoding RNAs (ncRNAs) defy the central dogma by representing a family of RNA molecules that are not translated into protein but can convey information encoded in their DNA. Elucidating the exact function of ncRNA has been a focus of discovery in the last decade and remains challenging. Nevertheless, the importance of understanding ncRNA is apparent since these molecules regulate gene expression at the transcriptional and post-transcriptional level exerting pleiotropic effects critical in development, oncogenesis, and immunity. NcRNAs have been referred to as “the dark matter of the nucleus”, and unraveling their role in physiologic and pathologic processes will provide vast opportunities for basic and translational research with the potential for significant therapeutic progress. Consequently, strong efforts are underway to exploit the therapeutic utility of ncRNA, some of which have been approved by the US Food and Drug Administration and the European Medicines Agency. The use of ncRNA therapeutics (or “vaccines” if defined as anti-disease agents) may result in improved curative strategies when used alone or in combination with existing treatments. This review will focus on the role of ncRNA therapeutics in prostatic carcinoma while exploring basic biological aspects of these molecules that represent about 97% of the transcriptome in humans.

## 1. Introduction

Noncoding RNAs (ncRNAs) represent a family of RNA molecules that are not translated into protein but execute a multitude of biological functions informing significant therapeutic potential. It is possible that some ncRNAs are nonfunctional products of spurious transcription, sometimes referred to as “junk RNA”. However, the exact number and function of ncRNAs are still a matter of debate and the focus of active research. It has been estimated that ncRNAs represent approximately 97% of the transcriptome, widely surpassing the amount of coding messenger RNAs (mRNAs). Approximately 30,000 molecules of ncRNAs have been identified, which may be functionally as important as proteins. Interestingly, it has been recognized that approximately 22% of ncRNAs have been misclassified and do encode small polypeptides, which may muddle translational research [1,2]. Overall, ncRNAs can be divided according to their length and function. Molecules longer than 200 nucleotides are referred to as long-intervening ncRNAs (lncRNAs or lincRNAs). They can be intronic or intergenic (lincRNAs) and play multifunctional roles regulating gene expression. Similar to mRNAs, lncRNAs are transcribed by RNA polymerase II, have a capped 5′ end, a polyadenylated 3′ end, and are processed by splicing. The nucleotide sequence of lncRNAs is not well conserved among species. However, their function is evolutionarily conserved, because lncRNAs with diverse sequences share a similar tertiary structure (hairpin) and bind to analogous proteins in different organisms [3]. In addition to proteins, lncRNAs can also directly bind to nucleic acids with bidirectionally (sense or antisense) base paring ability.

In contrast, the smaller ncRNA transcripts are usually about 20 nucleotides in length and represent about 2000 to 5000 molecules with evolutionarily well-conserved sequences that are processed by endonucleases. In common with lncRNAs, the location of smaller ncRNAs can be intergenic or intronic.

Functionally, ncRNAs are divided into homeostatic/housekeeping and regulatory types, although some molecules defy categorization. For example, circular RNAs (covalently closed molecules), which are usually lncRNAs that can, on occasion, encode proteins and be structurally shorter than 200 nucleotides long [4].

Well-known homeostatic ncRNAs include transfer RNAs (tRNAs) and ribosomal RNAs (rRNAs). tRNAs are short (~80 nucleotides) ncRNAs that ferry individual amino acids to ribosomes allowing protein translation. In contrast, rRNAs represent a specialized type of long ncRNAs (1500 to 3000 nucleotides in length) that constitute ~60% of the mass of the ribosomes and bind to riboproteins, contributing to the formation of the catalytic sites necessary for protein translation from mRNAs.

Regulatory ncRNAs have been functionally grouped into at least four main categories, sometimes displaying overlapping features: (1) splicing RNAs, (2) self-modifying RNAs, (3) transcriptional regulatory/gene silencing RNAs (all three usually belonging to the small ncRNA subclass), and (4) multifunctional gene-regulatory long RNAs, exemplified by lncRNAs. Splicing RNAs comprise small nuclear RNAs (snRNAs) that bind proteins in the spliceosome. Self-modifying RNAs represent small nucleolar RNAs (snoRNAs), which form part of the ribonuclear protein complex of the nucleolus and have functional similarity to guiding RNAs, serving to chemically modify other RNA molecules. The transcriptional regulatory/gene-silencing RNAs denote at least three types of molecules—namely, (a) small interfering RNAs (siRNAs), involved in gene silencing; (b) microRNAs (miRNAs), which represses mRNA translation by binding to the 3′ untranslated regions of mRNA, promoting its degradation; (c) PIWI-interacting RNAs (piRNAs), which interact with the piwi-subfamily of Argonaute proteins (a highly conserved family of RNA-binding proteins abbreviated as PIWI for the “P-element induced wimpy testis in Drosophila”), which predominantly silence transposable elements through both epigenetic, transcriptional and post-transcriptional mechanisms.

This overwhelming variety of ncRNA species, together with their abundance and rich overlapping functionality, suggests regulatory effects on almost every gene. For example, it has been estimated that in humans, one molecule of miRNA is able to bind with perfect or imperfect complementary base pairing to hundreds (100 to 500) of different mRNA molecules. Therefore, because more than 2000 mature miRNAs have been deposited in the miRNABase V22 [5], this implies that potentially every one of the ~20,000 genes in the human genome could be under miRNA regulation. Consequently, a tremendous effort to unravel the precise involvement of ncRNAs in biology is underway, to understand their crucial roles regulating development, immunity, and oncogenesis, and to explore novel therapeutic applications [6]. 

In this regard, ncRNA agents could act as immunomodulators or even therapeutic “vaccines”, when defined as medicines that contribute to eliminating or controlling disease, recognizing that so far the immunogenic potential of ncRNAs has not been exploited. Therefore, ncRNA therapeutics cannot be currently considered bona fide vaccines, and quotation marks will be used for clarification in the following sections. ncRNA “vaccines” can be mechanistically diverse, acting directly at a genetic or epigenetic level, or indirectly by modulating innate and adaptive immune responses to find and destroy harmful infectious agents and cancer cells. Modulating ncRNA represents an attractive strategy in oncologic therapy, as well as many other disciplines, and although RNA-based therapeutics have experienced significant prominence in the last 20 years, employing predominantly antisense oligonucleotides (ASOs) and small interfering RNAs (siRNAs), important hurdles need to be overcome. Several products have gained regulatory approval by the US Food and Drug Administration (FDA) or the European Medicines Agencies (EMA). However, trial results have been disappointing so far due to a combination of factors that include limited efficacy or significant toxicity. Alternative RNA products, such as lncRNAs and anti-miRNAs, are currently undergoing clinical evaluation, but importantly, other ncRNA species have not been extensively targeted to develop therapeutics yet.

In this review, we explore the potential for ncRNAs as therapeutics/“vaccines” in prostatic carcinoma (PC), since this disease remains a significant public health burden. Prostate cancer is the most frequently diagnosed nonskin cancer in the United States, representing approximately 6% of all cancer deaths (disproportionately affecting black men) and the second-most-common malignancy worldwide [7,8]. Even though the overall survival for PC is well above 90% [8], progression to incurable castration-resistant disease is relatively common and has been estimated to occur in 10 to 20% of cases during the first 5 years of follow-up [9]. Unfortunately, after castration resistance develops, the median specific survival time is ~1 year after the onset of metastasis [10]. Multiple therapeutic modalities are available for advanced PC, which have been comprehensively reviewed recently [11,12] and include hormone therapy, chemotherapy, immunotherapy, radiation and salvage prostatectomy, or various combinations of the aforementioned strategies. Despite significant therapeutic progress, metastatic PC remains incurable, and therefore, new treatments, including ncRNA therapeutics (which may be used alone or in combination with current options) are necessary. Furthermore, ncRNA may represent a multifunctional tool, acting directly by inhibiting tumor growth and indirectly by boosting anti-tumoral immune responses. Several lines of evidence indicate that ncRNAs modulate the immune response [13]. In fact, unintended immunogenicity elicited by ncRNAs represents a barrier to the applicability of these “vaccines” [14]. Our innate anti-viral defenses detect and target RNAs via pathogen-associated molecular pattern (PAMP) recognition molecules, such as Toll-like receptors (TLRs) and the retinoic acid-inducible gene 1 (RIG-1) receptors. Endosomal TLR3 and cytosolic RIG-1 sense double-stranded RNAs, while endosomal TLR7/8 sense single-stranded RNAs [15,16]. The sensing of foreign RNA leads to immune stimulation (cytokine release) inducing toxicity and possible shock. Chemical modification of RNA has been utilized to ameliorate this unwanted immunogenicity and could be tailored to preserve selective activation of signaling pathways that enhance anti-tumoral effects. Interestingly, it has recently been demonstrated that at least five miRNAs are associated with PC recurrence and metastasis [17], including miR-139, which induces interferon-beta expression in prostatic adenocarcinoma cell lines by transcriptionally activating genes downstream of RIG-1 (responsible for viral-induced interferon type-1 responses) [18]. These results suggest that miR-139 acts as an immune agonist eliciting anti-tumoral responses and can be modulated by ncRNA “vaccines” to treat PC. Similarly, the lncRNA HOX transcript antisense RNA (HOTAIR) has been shown to promote invasion and metastasis in PC [19] through inhibition of hepaCAM transcription, leading to activation of the MAPK pathway and highlighting new therapeutic opportunities. Unfortunately, despite the encouraging theoretical framework, none of the ncRNA therapeutics for PC has proven to be successful in the clinic so far. In the following sections, an examination of ncRNA therapeutics approved by regulatory agencies to treat PC will be undertaken, focusing on salient aspects that may illuminate future success.

## 2. Noncoding RNA Therapeutics Approved by Regulatory Agencies

Several excellent reviews have recently covered a variety of strategies to manipulate the medicinal potential of ncRNAs by using small interfering RNAs (siRNAs), antisense oligonucleotides (ASOs), short hairpin RNAs (shRNAs), anti-microRNAs (anti-miRNAs), miRNA mimics, miRNA sponges, therapeutic circular RNAs (cirRNAs), and CRISPR-Cas9-based gene editing [6,20,21]. The value of developing therapeutic targeting of ncRNA, while obvious, faces significant obstacles associated with specificity, delivery, and tolerability. Some clinical trials have been stopped, mainly due to lack of efficacy, and it remains uncertain if proper delivery was achieved. In addition, intolerable side effects related to activation of the immune system have promoted the termination of clinical trials. Toxicity is commonly related to the recognition of RNA as a foreign antigen by TLRs, which activate the transcription factor NFkB, leading to cytokine storm [22,23]. Nevertheless, this intrinsic ability of foreign ncRNA to elicit immune responses could be harnessed to avert tumoral immune escape and increase tolerability of ncRNA “vaccines”. Promising advances in this direction have been achieved with other RNA therapeutics, based on breakthroughs in immunology research [24,25]. Therefore, ncRNA could be deployed as a double-edged sword to modulate immune activation, allowing only desirable immunotherapeutic effects while simultaneously synergizing conventional anti-oncogenic chemotherapy. For instance, in the last decade, several studies have shown that intercellular communication can be mediated by nanosized extracellular vesicles (exosomes), which are present in all human body fluids and are composed of bilayer lipid membranes containing diverse cargo molecules (proteins, lipids, ncRNAs, and DNAs) [26]. The release of exosomal content into the cytoplasm of recipient cells after cell membrane fusion has been shown to alter protein expression in the recipient cells, regulating adaptive immune responses to microbes and tumors [27,28]. In addition, it has been specifically shown that ncRNA cargo in the exomes may alter the tumor microenvironment and macrophage function [29]. Recent studies have also addressed the role of exosomal ncRNAs in PC cell lines. Exosomal delivery of miR-26a inhibited metastasis and tumor growth in a PC mouse xenograft model [30]. Another report found that exosomal long lncRNA HOXD-AS1 was upregulated in the serum of patients with metastatic PC, suggesting that it may promote tumorigenesis by acting on the miR-361-5P/FOXM1 axis [31]. Collectively, these data reaffirm the possibility of developing tailored ncRNA therapeutics with reduced toxicity and improved specificity by exploiting recent innovations in chemical engineering.

Despite the rapidly evolving refinements in precision medicine, ncRNA “vaccine” development remains daunting. Many different ncRNA therapeutic products are in the pipeline, but only a few have been approved by the FDA and/or the EMA. Most of them were designed to treat monogenic inherited diseases by using siRNAs or ASOs that would specifically downregulate wild-type genes or alter pathological splicing of mutated genes restoring functionality. However, none of these tools have been approved to treat carcinomas due, in part, to the complex multigenic nature of malignancy. Nevertheless, several ncRNA therapeutics are in phase II or III clinical development, which include different molecules such as miRNA mimics and anti-miRNAs [6]. Of these, at least a few are intended for carcinomas such as pancreatic, nonsmall-cell lung, and colorectal.

Notably, three products have been deployed in clinical trials for advanced PC (Table 1). Apatorsen (OGX-427), attempted to target castration-resistant PC in a phase II trial (NCT01120470). Apatorsen is a second-generation ASO that targets the cytoprotective Hsp27, a chaperone in the heat shock family of proteins. Downregulation of Hsp27 by apatorsen is expected to enhance the sensitivity of prostatic tumors to cytotoxic agents [32]. In the NCT01120470 randomized study, adding apatorsen to low-dose prednisone did not change the progression-free survival when compared with prednisone alone in 74 patients with metastatic PC or pelvic recurrence who were chemotherapy naïve. However, a significant decrease in prostate specific antigen (PSA) was observed in the apatorsen plus prednisone arm [33]. Since this study used an endpoint (nonprogression at 12 weeks) not currently endorsed by the Prostate Cancer Working Group criteria [34], further research on apatorsen is necessary for optimal standardization [35]. However, these results demonstrated the potential therapeutic benefit of targeting Hsp27 with an ASO in PC and spearheaded further research to develop alternative strategies to treat advanced disease. Reassuringly, similar conclusions were reached in another clinical trial (NCT01454089) analyzing the treatment of advanced urothelial carcinoma using apatorsen in combination with docetaxel. In this phase II trial, patients randomized to apatorsen and docetaxel had improved overall survival, compared with docetaxel alone, in metastatic or relapsed urothelial carcinoma [36]. Conversely, the NCT01829113 double-blinded, randomized clinical trial for untreated metastatic nonsquamous/nonsmall-cell lung carcinoma found no benefit from the addition of apatorsen to carboplatin and pemetrexed in 155 patients, attesting to the histological specificity of Hsp27 inhibition for at least prostatic and urothelial carcinomas. Notably, these three clinical trials using apatorsen also established the acceptable tolerability of this ncRNA agent [37]. Finally, the trial NCT02423590 was approved to determine the effect of apatorsen in combination with first-line chemotherapy (gemcitabine/carboplatin) in advanced squamous cell lung carcinoma, and the results are still unavailable to the public.

Other genes have also been targeted by ncRNA therapeutics in clinical trials for advanced PC. The AFFINITY phase III trial employed the ASO custirsen (OGX-011, and CC-8490) to downregulate Clusterin, a heat shock protein that regulates apoptosis. Interestingly, the results contradicted previous clinical trials since no significant survival benefits could be demonstrated in patients with metastatic castration-resistant PC [38]. The trial design included 635 men with similar demographics randomized into two arms with the treatment arm receiving chemotherapy (cabazitaxel and prednisone) and custirsen, while the control arm received only chemotherapy. This ASO was well-tolerated and severe adverse effects (seen in up to approximately 22% of patients) were equally distributed among the two arms of the study. Clusterin is a complex protein, with three alternatively spliced isoforms that have different cellular/extracellular localization (nuclear, cytoplasmic, and secreted/extracellular), which determines its function [39]. The nuclear form of clusterin plays a pro-apoptotic role by displacing BAX from its complex with the DNA-binding protein Ku70. The released BAX then inserts into the outer membrane of the mitochondria, creating a permeability pore that facilitates cytochrome c release, leading to caspase activation, which triggers the mitochondrial cell death program [40,41]. In contrast, the cytosolic and extracellular isoforms of Clusterin have anti-apoptotic functions by inhibiting BAX and p53 [42,43]. Taken together with the possibility of cell lineage-dependent functional variability, complex regulation of these three distinct isoforms of Clusterin may explain, in part, the disappointing results of this clinical trial. A promising randomized phase II clinical trial was established to evaluate the effect of inhibiting the anti-apoptotic proto-oncogene Bcl-2 using the ASO oblimersen sodium in addition to docetaxel also in castration-resistant PC [44]. The endpoint selected was a reduction of PSA > 30%, which was not achieved in any arm of the study, indicating that oblimersen was not beneficial in this selected cohort of 111 patients. Interestingly, the expression of Bcl-2 was not analyzed, raising the possibility of a delivery failure. Future clinical trials with oblimersen selecting tumors with known overexpression of BCL2 or with a more granular evaluation of delivery issues may be informative. However, major toxicity may be an obstacle to using oblimersen since significant adverse events (mainly myelosuppression) were observed in 40.7% of patients treated with oblimersen and docetaxel in combination but only in 22.8% of those treated with docetaxel alone. Considerable attention has been given to investigating the utilization of the miRNA-34a (miR-34a) as a target of ncRNA “vaccines”, because it is considered a tumor suppressor that acts in synergy with p53 and may be downregulated in several malignant neoplasms from diverse origins, including prostate, bladder, lung, breast, gastrointestinal, pancreas, liver, head and neck, ovary, bone and hematopoietic [45,46]. MiR-34a is transcriptionally upregulated by p53 [47] and also inhibits the epithelial–mesenchymal transition associated with overexpression of CD44 and metastatic behavior of cancer stem cells [48,49]. Interestingly, miR-34a has been shown to directly inhibit tumor expression of the adhesion molecule CD44, which interacts with the extracellular matrix promoting migration and invasion of tumor cells, which is characteristic of metastatic behavior [50]. Therefore, a phase I clinical trial using MRX34 (a liposomal mimic of microRNA-34a) was established for advanced solid tumors as a multicenter study (NCT01829971). Unfortunately, this study was closed prematurely due to serious adverse effects including four deaths in a cohort of 85 patients. However, the study provided additional proof of concept for using ncRNA therapeutics, since the targeted genes were appropriately modulated, and satisfactory cytoplasmic delivery of MRX34 was demonstrated in the treated patient’s tumors by detecting increased miR-34a expression using chromogenic in situ hybridizations [51]. MRX34 was administered intravenously along with oral dexamethasone, and the study concluded by emphasizing the necessity to better understand the biology of immune stimulation induced by double-stranded RNA pharmacomimetics (such as MRX34) before implementation of new clinical trials in humans. Surprisingly, pre-clinical animal trials did not predict the observed toxicity, which also included immune hepatitis, myelosuppression, fever/chills, and dyspnea.

## 3. Circumventing Obstacles to Efficacious Noncoding RNA Therapeutics

The immense therapeutic potential of ncRNA “vaccines” remains still unrealized, partly due to toxicity and delivery issues, which could be tackled with evolving innovation in the field [6]. As alluded in the preceding sections, the immunogenicity of foreign RNA accounts for most toxic effects of ncRNA therapeutics, which activate TLR signaling and myeloid differentiation factor 88 (MYD88), ultimately terminating in excessive release of cytokines (IL-6, IL-8, IL-12, and TNF) and IFN type I [52]. Several approaches have been introduced to increase the tolerability of ncRNA. As double-stranded RNA is less potent at activating the immune system [53], all ncRNA therapeutics currently in use employ single-stranded RNA. In addition, chemical modification of the known immunogenic sequences in ncRNA (for example GU-rich sequences such as 5′-UGUGU-3′ or 5′-GUCCUUCAA-3′) has been engineered using various strategies, including 2′-ribose modifications of uridine or guanosine nucleosides that can abrogate TLR stimulation [54]. Similarly, preservation of the two nucleotide-long 3′-overhangs (the natural product generated during the biogenesis of miRNA by the enzyme Dicer) present in endogenous miRNAs has been shown to decrease immune activation and was engineered in 5′-triphosphate-modified siRNAs [55,56]. Despite these and several other advances in RNA chemical engineering, the clinical trials using modified agents have been disappointing due to unacceptable toxicity related to immune stimulation. For example, flu-like symptoms were still seen with a modified siRNA (PRO-040201) to downregulate Apolipoprotein B in a clinical trial to treat hypercholesterolemia (NCT00927459).

An alternative method to decrease the unwanted immunogenicity of ncRNA “vaccines” is exemplified by third-generation modifications, such as phosphoramidate morpholino oligomers (PMOs or simply morpholinos) that should not activate TLR signaling [57]. PMOs are small (usually 25 nucleotides long) synthetic nucleic acid molecules that bind to native single-stranded RNAs or DNAs by standard base pairing. However, since the bases of morpholinos are bound to methylene morpholine rings linked by phosphorodiamidate (instead of phosphates present in natural nucleic acids), PMOs are not charged at physiologic pH, and the morpholino–RNA complexes are not degraded. Therefore, stimulation of TLRs by RNA degradation products should not occur, and the morpholinos could be used for the selective knockdown of genes by a mechanism analogous to siRNAs and ASOs. For example, eteplirsen is an FDA-approved translation-blocking morpholino ASO used in Duchenne muscular dystrophy (DMD) to induce skipping of the mutated exon in dystrophin, which allows the translation of an almost full-length functional form of the protein encoded by the DMD gene. Eteplirsen binds to exon 51 of dystrophin pre-mRNA, preventing incorporation of the pathogenic missense mutation into mature mRNA, circumventing pre-mature termination of the mutated DMD gene [58]. Eteplirsen has been well tolerated in clinical trials [59], which indicates that the chemical modification of ncRNA agents may solve their toxic side effects [60].

Several additional strategies are being explored to overcome excessive immunogenic effects of ncRNA agents, including screening for possible TLR activation before deployment, and using “tiny” antisense RNA, metronomic miRNA therapy, and combinatorial RNA therapeutics, as has been thoroughly reviewed recently [6]. Interestingly, none of these newer techniques have been attempted in PC yet, underscoring exciting future opportunities for clinical trials. At present, it remains unclear whether ncRNA “vaccines” have been effective by directly modulating neoplastic gene expression, or indirectly by triggering anti-tumoral immune responses. What has become clear is that achieving optimal regulation of the immunogenicity induced by ncRNA therapeutics would require a multidisciplinary effort to further capitalize on this promising technology.

Another prominent obstacle for optimal efficacy of ncRNA products is controlling their specific delivery.

The most common cause of termination of ncRNA “vaccine” clinical trials has been the lack of efficacy, which may be multifactorial including stability and delivery issues. Efforts to improve the delivery of ncRNA therapeutics must consider both the delivery of these agents into the targeted organ, as well as access to the cytoplasm of the appropriate cell type [61]. Various strategies are being perfected to optimize proper transport systems, including vectors (liposomes, polymeric nanoparticles, and viruses) and conjugate delivery methods, which have been recently reviewed elsewhere and are beyond the scope of this article [62]. The natural instability of RNAs due to the presence of a 2′-OH group susceptible to base-catalyzed hydrolysis and the prevalence of ribonucleases (RNAses) prompted the development of chemical modifications of RNA therapeutics to promote stability. First- and second-generation alterations enhanced resistance to RNAses and interactions with proteins to deter degradation [63].

Third-generation antisense oligonucleotides (for example, locked nucleic acid, peptide nucleic acid, and morpholino phosphoramidates) utilized modifications of the furanose ring and the phosphodiester bonds to improve resistance to RNAses but also to target affinity and pharmacokinetic profiles [64], as described in preceding paragraphs.

Interestingly, local delivery schemes using lipid nanoparticle-like systems have shown success in PC cell lines [65] and in diverse animal models [66,67], resulting in FDA approval. For example, patisiran, a siRNA-therapy used to treat amyloidosis by silencing transthyretin [68] and the previously described liposomal mimic of miR-34a, MRX34 [51]. Novel conjugates of ncRNA with various molecules (polymers and antibodies) to improve delivery also represent promising arsenals now in early clinical trials or preclinical development. However, none of these delivery systems has produced efficacious results in PC to date.

## 4. Conclusions

In conclusion, we reviewed exciting and growing applications of ncRNA therapeutics, focusing on clinical trials and future prospects for PC. The results of the trials in PC have been so far contradictory and plagued with issues related to toxicity and suboptimal delivery. However, substantial scientific advances reaffirm the potential of ncRNA therapeutics for the treatment of a multitude of diseases, including PC. The fact that one ncRNA molecule controls numerous different genes in an amplifying effect reinforces the therapeutic value of these agents.

Third-generation chemical modification of ncRNA “vaccines” has achieved a level of sophistication that ensures acceptable tolerability and pharmacokinetic profiles. New delivery systems, including conjugates with nanopolymers and/or specific antibodies, highlight the promise of precise targeting. To date, most of the clinical trials have used anti-miRNA ASO (antagomiRs) and siRNA, which leaves the field wide open for development and experimentation with other ncRNA therapeutics in the future. Ample opportunities to explore ncRNA species exist that have not been used in the clinic, such as miRNA sponges (linear or circular artificial RNA molecules able to simultaneously inhibit more than one species of miRNA) [69,70] or miRNA-masking ASOs (able to block the access of native miRNA to the target mRNA by annealing to the latter in a sequence-specific manner) [71]. Furthermore, some types of lncRNAs, such as circular RNAs or natural antisense transcripts, may represent innovative therapeutic opportunities, which have only recently gained attention for clinical trials [6]. The realization of the potential of ncRNA therapeutics will require a multidisciplinary approach combining scientific advances in the fields of molecular biology, immunology, chemistry (nanotechnology), and pharmacology, coupled with translational research in various clinical disciplines including oncology. The ideal ncRNA product should act specifically on one or various genetic pathways in the appropriate tissue type without eliciting intolerable toxicity related to an exaggerated immune response. Encouraging creative solutions are emerging in ncRNA therapeutics, which allow cautious optimism and certainly will foster additional multidisciplinary research.

In summary, the field of ncRNA therapeutics is reaching maturity and offers tremendous hope to deliver a revolutionary change in the treatment of carcinoma and many other maladies.

## Figures and Tables

**Table 1:** Clinical trials for advanced prostatic carcinoma using ncRNA therapeutics.

Trial Title	Therapeutic (Type) and MOA	Characteristics	Regimen	Major Outcomes
A Randomized Phase II Study of OGX-427 (a Second-Generation Antisense Oligonucleotide to Heat Shock Protein-27) in Patients with Castration-Resistant Prostate Cancer Who Have Not Previously Received Chemotherapy for Metastatic Disease. (NCT01120470)	Apatorsen/OGX-427 (a second-generation ASO) targets cytoprotective Hsp27. Downregulation of Hsp27 is expected to enhance sensitivity to cytotoxic agents.	74 patients were randomized to receive apatorsen + prednisone (*n* = 36) or prednisone alone (*n* = 38). The primary endpoint was disease progression at 12 weeks.	Three loading doses at 600 mg IV within the first 10 days of initiating treatment, followed by weekly doses of 1000 mg IV up to 12 weeks.	Apatorsen + prednisone produced a significant PSA decline but did not change the proportion of CRPC patients without disease progression at 12 weeks, compared with prednisone alone.
A Randomized Phase III Study Comparing Cabazitaxel/Prednisone in Combination with Custirsen (OGX-011) to Cabazitaxel/Prednisone for Second-Line Chemotherapy in Men with Metastatic Castrate-Resistant Prostate Cancer (AFFINITY) (NCT01578655)	Custirsen (ASO) downregulates Clusterin. Clusterin (a cytoprotective heat shock protein) regulates apoptosis and is upregulated by chemotherapy.	635 patients were randomized. Co-primary objectives were to evaluate overall survival (OS) in patients receiving Cbz/P/C (*n* = 317) versus Cbz/P (*n* = 318) alone.	21-day cycles of 25 mg/m2 IV Cbz on day 1 with 10 mg oral P daily with or without 640 mg IV of C on days 1, 8, and 15 (plus 3 prior loading doses) until disease progression, unacceptable toxicity, or 10 cycles obtained.	No significant survival benefits were demonstrated.
Randomized Phase II Trial of Docetaxel (Taxotere) and Oblimersen (Antisense Oligonucleotide Directed to BCL-2) versus Taxotere Alone in Patients with Hormone-Refractory Prostate Cancer (NCT00085228)	Oblimersen (ASO) selectively downregulates Bcl-2 (anti-apoptotic proto-oncogene) expression.	115 Chemotherapy naive patients were randomized to receive docetaxel + oblimersen (*n* = 58) or docetaxel (*n* = 57) alone. Biologic anti-tumor activity (based on PSA response: Bubley Criteria)	Docetaxel 75 mg/m^2^ on day 1 or oblimersen 7 mg/kg/day continuous IV infusion on days 1–7 with docetaxel 75 mg/m^2^ on day 5 every 3 weeks for ≤12 cycles. Patients in the docetaxel group received a median of eight cycles and those in the docetaxel + oblimersen group received a median of six cycles	The selected endpoint (reduction of PSA > 30%) was not achieved in any arm of the study, indicating that oblimersen was not beneficial in this selected cohort, but Bcl-2 expression was not analyzed.

MOA = mechanism of action, Cbz = cabazitaxel, P = prednisone, C = custirsen.

## Data Availability

Not applicable.
